# Estimating program coverage in the treatment of severe acute malnutrition: a comparative analysis of the validity and operational feasibility of two methods

**DOI:** 10.1186/s12963-018-0167-3

**Published:** 2018-07-03

**Authors:** Sheila Isanaka, Bethany L. Hedt-Gauthier, Rebecca F. Grais, Ben G. S. Allen

**Affiliations:** 1 Department of Research, Epicentre, 8 rue Saint Sabin, 75011 Paris, France; 2 Departments of Nutrition and Global Health and Population, Harvard T.H. Chan School of Public Health, Boston, USA; 3 Department of Global Health and Social Medicine, Harvard Medical School, Boston, USA; 4 Department of Biostatistics, Harvard School of Public Health, Boston, USA; 5 Norwich, UK

**Keywords:** Bayesian conjugate analysis, Cluster survey, Coverage, Severe acute malnutrition, SQUEAC, Therapeutic feeding program

## Abstract

**Background:**

Many health programs can assess coverage using standardized cluster survey methods, but estimating the coverage of nutrition programs presents a special challenge due to low disease prevalence. Used since 2012, the Semi-Quantitative Evaluation of Access and Coverage (SQUEAC) employs both qualitative and quantitative methods to identify key barriers to access and estimate coverage of therapeutic feeding programs. While the tool has been increasingly used in programs, the validity of certain methodological elements has been the subject of debate.

**Methods:**

We conducted a study comparing a SQUEAC conjugate Bayesian analysis to a two-stage cluster survey estimating the coverage of a therapeutic feeding program in Niger in 2016.

**Results:**

We found that the coverage estimate from the conjugate Bayesian analysis was sensitive to the prior estimation. With the exception of prior estimates produced by an external support team, all prior estimates resulted in a conflict with the likelihood result, excluding interpretation of the final coverage estimate. Allowing for increased uncertainty around the prior estimate did not materially affect conclusions.

**Conclusion:**

SQUEAC is a demanding analytical method requiring both qualitative and quantitative data collection and synthesis to identify program barriers and estimate coverage. If the necessary technical capacity is not available to objectively specify an accurate prior for a conjugate Bayesian analysis, alternatives, such as a two-stage cluster survey or a larger likelihood survey, may be considered to ensure valid coverage estimation.

**Trial registration:**

NCT03280082. Retrospectively registered on September 12, 2017.

**Electronic supplementary material:**

The online version of this article (10.1186/s12963-018-0167-3) contains supplementary material, which is available to authorized users.

## Key messages

- Valid estimates of nutrition program coverage in low- and middle-income countries are needed to assess program performance and inform allocation of resources and policy.
- The Semi-Quantitative Evaluation of Access and Coverage (SQUEAC) methodology represents a step forward in coverage assessment of therapeutic feeding programs by providing a routine means by which to simultaneously identify barriers to accessing care and estimate program coverage.
- The SQUEAC conjugate Bayesian analysis proposed for coverage estimation can be a technically demanding method that depends on accuracy of the prior estimate. Methods to develop a final prior estimate have been suggested, but there is little evidence regarding the validity of their application in capacity-limited settings. Subjective and overly certain prior estimates produced from these methods may render the final coverage estimate invalid. The appropriate technical capacity is necessary during prior estimation to ensure informative results. More research to review and validate methods to produce accurate prior estimates may be needed.
- Program managers should decide on the appropriate method to be used based on information needed and availability of appropriate capacity to implement the SQUEAC method.

## Background

Seventeen million children are estimated to suffer from severe acute malnutrition (SAM) each year [1] and experience a nine-fold increase in the risk of death [2]. Specialized treatment exists, and community-based management has been shown to be both effective and cost-effective [3–7]. The public health impact of treatment, however, is a function of both effectiveness and coverage. Low levels of program coverage, where the proportion of children receiving treatment among those in need is low, can reduce program quality and impact.

Coverage of many health programs can be assessed using cluster survey methods, but coverage estimates for nutrition programs present special challenges due to the low prevalence of disease and often limited resources for data collection. Since 2012, the Semi-Quantitative Evaluation of Access and Coverage (SQUEAC) methodology has been widely used to assess the coverage of SAM treatment programs [8]. In contrast to other survey methods, including two-stage cluster surveys or centric systematic area sampling, SQUEAC relies on a mix of qualitative and quantitative methods to estimate coverage while also identifying key barriers to access. It is thought to provide an operational advantage over traditional methods as primary data collection can be quick and relatively inexpensive, allowing for frequent evaluation by program staff. To keep the burden of primary data collection low, coverage estimates are obtained using a conjugate Bayesian analysis, in which information of coverage from routine program data and qualitative information are updated with primary data from a relatively small sample to construct an overall posterior estimate.

While SQUEAC was developed as a practical, low-cost tool for programs to assess the quality of therapeutic feeding programs, questions have been raised over the optimal balance between validity and operational feasibility of the conjugate Bayesian analysis [9]. In particular, concerns have been raised that the methods underlying the formulation of the prior used in the conjugate Bayesian analysis can be too subjective. This subjectivity, along with overly optimistic certainty when forming the prior, could lead to conflicts between the prior and the likelihood data, rendering the final posterior estimate invalid. To better understand the strengths and potential challenges of the SQUEAC method, we assessed the coverage of a therapeutic feeding program in Niger in 2016, using both two-stage cluster survey methods and SQUEAC, with multiple approaches to forming the prior. Here, we describe the results and operational feasibility of these methods.

## Methods

### Study setting

This study was conducted in the Madaoua health district in the Tahaoua region of southern Niger. Madaoua is situated in the Sahel region and is characterized as largely agricultural with a high seasonal burden of acute malnutrition. In support of the Ministry of Health, Médecins Sans Frontières (MSF) has been operational in the region since 2006, supporting one inpatient and six outpatient centers for the management of SAM with community surveillance and outreach teams operational in 80 villages.

### SQUEAC methodology

The SQUEAC methodology was designed to be used on a regular basis to monitor program performance, identify barriers to service access and uptake, and estimate coverage [8]. It uses a flexible mix of routine program data and surveys and qualitative data (Stage 1 and 2 data) to construct a prior. This is combined with primary likelihood data (Stage 3 data) using a beta-binomial conjugate Bayesian analysis to obtain a final coverage estimate and a posterior probability density. More details are available in the Additional file 1.

### Study procedures and statistical analysis

#### Prior estimation

Prior estimates of coverage were estimated using five recommended methods [8] and three participant sources, including the program team (two groups of five to six people), caregivers/beneficiaries identified at a treatment site (five groups of two to four women) and an external support team (two consultants from the Coverage Monitoring Network and Epicentre) (further detail provided in Additional file 1). 1) The weighted score approach asked each participant group to synthesize available information into a set of positive (boosters) and negative (barriers) factors affecting access and coverage. Each factor was listed and weighted by importance by the participants. 2) A simple scoring approach, in which the same set of boosters and barriers listed by the group was assigned the same weight, was also used. 3) The histogram of belief was estimated, in which all possible values of coverage value (0 to 100%, x-axis) were discussed and the level of belief of whether each value was likely to be true (y-axis) was collectively determined to create a histogram of belief of coverage. 4) The product of program performance method multiplied quantitative estimates of various program indicators produced during Stage 1 and deducted from the theoretical maximum coverage of 100%. 5) A previous SQUEAC assessment was conducted in the same program area in 2013: the final coverage was reported to be 82.6% (95% CI: 76.7–88.5%) using the single coverage estimator [10].

It is recommended to triangulate credible prior information to develop a single, final prior estimate for use in the conjugate Bayesian analysis [8]. We therefore combined individual prior estimates from multiple methods and sources using a simple unweighted mean in three scenarios. *Scenario 1* represented a “broad program staff implementation” that included all methods for prior estimation but did not include estimates from the external support team. *Scenario 2* represented a more “basic program staff implementation” that did not include weighted scores, which may be considered to be more time- and resource-intensive methods that are less likely to be utilized in constrained settings, nor estimates from the external support team. *Scenarios 1* and *Scenario 2* are likely to represent common field conditions, where recently trained program staff (eg, without previous experience with SQUEAC or coverage estimation) weight boosters and barriers to develop a prior without input from experts. *Scenario 3* represents the case of “external implementation” when expert opinion is available to weight boosters and barriers and included only prior estimates developed by the external support team.

Current guidance suggests greater uncertainty be assigned to the prior estimate when the position of the prior mode is not certain, or the priors from various methods and sources show high variability. We therefore allowed standard (± 25%) and greater uncertainty (± 35%) for each prior estimate.

#### Sampling and data collection for SQUEAC likelihood survey (Stage 3 data) and two-stage cluster survey

Village selection was made using spatial stratified systematic sampling. A complete list of villages was sorted by geographically delimited health area, and villages were selected systematically to yield a reasonably even spatial sample across health areas. Within the selected villages all SAM cases were found using exhaustive active and adaptive case-finding, a census sampling method (further detail in Additional file 1).

This study aimed to compare coverage estimates from a SQUEAC versus a two-stage cluster survey. It is generally expected that the sample size required for a two-stage cluster survey will be larger than the sample sizes required for a SQUEAC of the same precision, as the conjugate Bayesian analysis leverages prior information to reduce the required sample size. We therefore sampled a sufficient number of cases/villages to power the two-stage cluster survey with adequate precision and randomly drew a subset of these villages for the SQUEAC analyses. Methods for village selection and case-finding at the village level were thus identical between analytical approaches.

The required sample size (SAM cases) in a two-stage cluster survey was estimated using Eq. 1, where p = the most likely estimate of coverage and type 1 error = 0.05. In order to achieve a precision of ±10% and assuming the most conservative *p* = 50%, 96 SAM cases were required. It was estimated that 46 villages were required to find 96 SAM cases (further detail in Additional file 1).

1 $$ n=\frac{p\times \left(1-p\right)}{{\left( precision\div 1.96\right)}^2} $$

The sample size required for the SQUEAC likelihood survey (Stage 3) depended on the prior distribution (described by the mode and two shape parameters α and β) and the precision (e.g., ± 10%) desired for the posterior estimate (Eq. 2). Depending on the prior distribution, we estimated that 62 to 81 SAM cases (Eq. 2), and thus 30 to 39 villages, were required for the SQUEAC.

2 $$ n=\left\lceil \frac{mode_{prior}\times \left(1-{mode}_{prior}\right)}{{\left( precision\div 1.96\right)}^2}-\left(\ {\alpha}_{prior}+{\beta}_{prior}-2\right)\right\rceil \kern7em $$

#### Statistical analysis

Single coverage was estimated using the observed number of uncovered SAM cases, observed number of covered recovering cases, and an estimate of uncovered recovering cases (further detail in Additional file 1). Single coverage is currently recommended over the use of period and point coverage estimates, as it allows for consideration of both covered and uncovered recovering cases [11].

To estimate single coverage for the SQUEAC, we drew the required subset from the 46 villages surveyed for two-stage cluster survey at random and without replacement from the larger set. Point estimates and 95% confidence intervals were taken as the median value and the 2.5th and 97.5th percentiles, respectively, from 10,000 draws.

Conflicts, which manifest as little or no overlap between the distributions of the prior and the likelihood, invalidate posterior results from the conjugate Bayesian analysis. In case of a conflict, coverage estimates may only be calculated from likelihood survey data and final estimates will be less precise due the smaller likelihood sample size. The presence of a conflict between each prior and the likelihood probability density was assessed using a Z test.

## Results

**Fig. 1:**
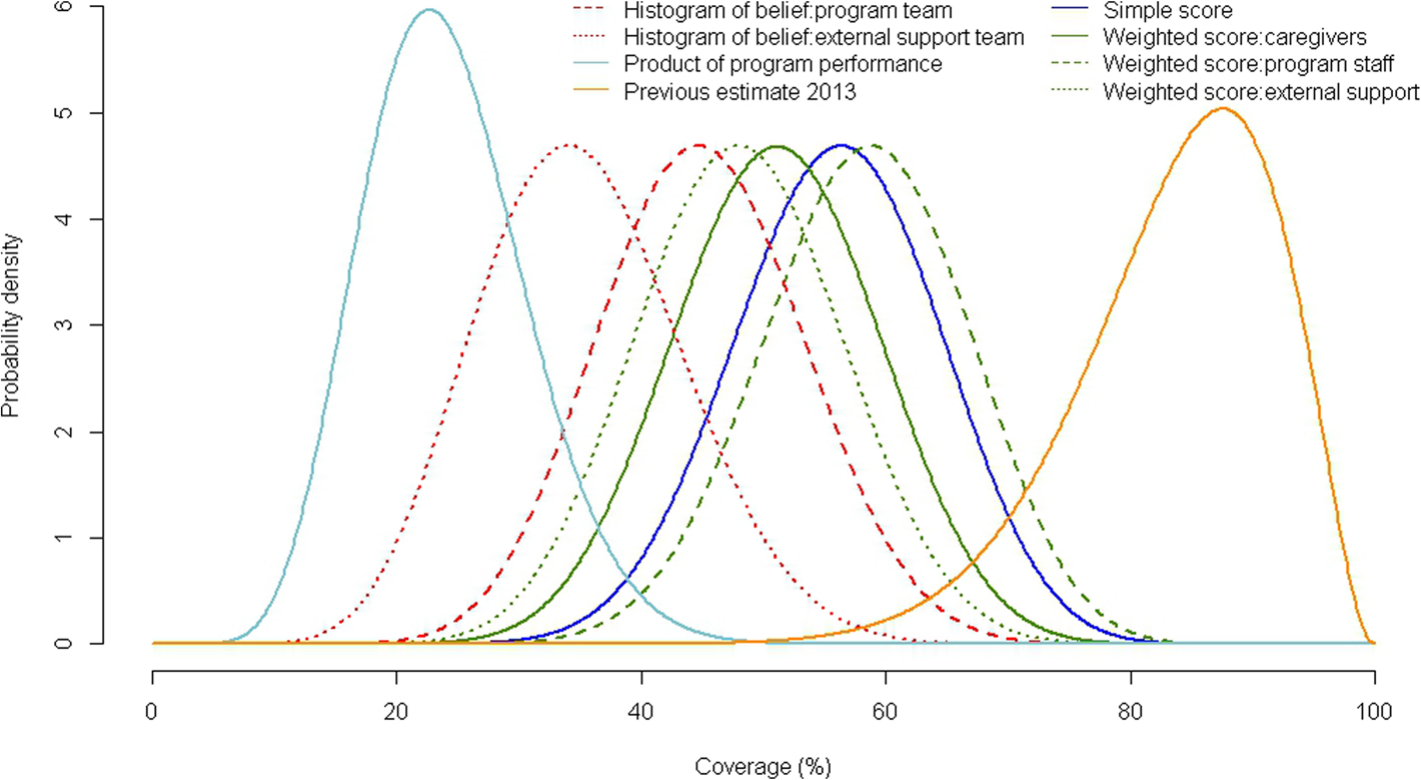
Distribution of eight prior estimates, by exercise and participant source.

Stage 1 quantitative and qualitative data collection and analysis yielded a set of 15 boosters and 11 barriers affecting access and coverage. Simple and weighted scores for each factor are shown in Table 1. Individual prior estimates produced using all five exercises and three participants groups are shown in Table 2 and Fig. 1. We found little differentiation between the individual prior estimates calculated as a simple versus weighted score from the program staff (56% versus 58%), while individual prior estimates calculated using the weighted scores of caregivers tended to be higher (62%) and those calculated using weighted scores from the external support team tended to be lower (48%). The histogram of belief exercise yielded lower prior estimates than either the simple or weighted score exercise, with the histogram generated by the external support team was lower than the histogram produced by the program staff (35% versus 45%). The product of program performance yielded the lowest individual prior estimate (24%) and the previous SQUEAC assessment the highest (84%). The unweighted means of the individual prior estimates included in each scenario are shown in Table 3.

**Table 1:** Boosters and barriers to access and coverage found during Stage 1 and 2, with scoring for each method

	Weighted scores^a^			Simple score
	Caregivers	Program staff	External support team	
Boosters				
Good use of the health post where screening and referral of SAM cases takes place	3	3	1	3
A preference for treatment with ready-to-use therapeutic foods from the health centers	3	3	1	3
Frequent sensitization of caregivers at health centers, which improves retention	2	2.5	1	3
Sharing of information on the program by caregivers who are (or were) in the program	3	2.5	2	3
Information on malnutrition and community-based management of acute malnutrition diffused by local radio	3	1.5	1	3
Sensitization during home visits by community nutrition volunteers supported by NGO	3	2	1	3
Knowledge on malnutrition among the community	3	3	2	3
Knowledge on the existence of CMAM services among the community	3	3	3	3
Knowledge and appreciation of free health care that encourages presentation at health centers	3	3	2	3
Good management of stock and continuous service delivery	2	3	1	3
Screening at village level by MSF surveillance team	3	2	2	3
Screening at village level by NGO-supported community nutrition volunteers	3	2	1	3
System in place for following up absent and defaulting cases	3	2	1	3
Service has a positive reputation due to the good behavior of staff and a calm and efficient management of the CMAM sites	3	3	2	3
Caregivers have the support of their husbands, family, and/or the community that encourages them to go to the health center	3	2	2	3
Boosters total	43	37.5	23	45
Barriers				
Poor condition of the roads between the village and the health center	3	1.5	3	3
Distance between the village and the health center is too long	3	2	3	3
A lack of means for making the journey to the health center (availability of finances or transport)	3	1.5	3	3
Screening by MSF teams is done at a central point in the villages and not door-to-door	2	2.5	3	3
Refusal of husband or family, or lack of support to search for treatment	1	1.5	1	3
Insufficient staff numbers to ensure an efficient management of CMAM services at health center	1	2	1	3
Perception that the caregiver does not have the time and therefore does not prioritize visiting the health center	1	3	3	3
Alternative health-seeking behavior (traditional health practitioner or pharmacy)	1	3	3	3
Lack of knowledge on CMAM services among the community	1	1	3	3
Lack of knowledge on malnutrition among the community	2	1	2	3
Lack of knowledge that children can be readmitted	2	2	2	3
Barriers total	20	21	27	33

^a^1 point for low importance for access and coverage, and 3 points for high importance on access and coverage

**Table 2:** Individual prior estimates contributing to three scenarios of the SQUEAC conjugate Bayesian analysis

Method	Source	Barriers score	Boosters score	Calculation	Prior estimate	Scenario 1	Scenario 2	Scenario 3
Simple scoring	–	45	33	$\frac{33+\left(100-45\right)}{2}$	56%	✓	✓	
Weighted scoring	Caregivers	43	20	$\frac{20+\left(100-43\right)}{2}$	62%	✓		
Weighted scoring	Program staff	37.5	21	$\frac{21+\left(100-37.5\right)}{2}$	58%	✓		
Weighted scoring	External support team	23	27	$\frac{27+\left(100-23\right)}{2}$	48%			✓
Histogram of belief	Program staff	–	–	–	45%	✓	✓	
Histogram of belief	External support team	–	–	–	35%			✓
Product of program performance	Stage 1 data	–	–	100 % × 72.5 % × 45.4 % × 86 % × 88 % × 99%	24%	✓	✓	
Previous assessment	SQUEAC report 2013	–	–	–	84%	✓	✓

**Table 3:** Prior and posterior estimates for cluster sampling coverage survey and SQUEAC conjugate Bayesian analysis

	Final prior coverage estimate	Likelihood coverage estimate		Posterior coverage estimate	Strength of evidence for conflict^d^
Scenario for prior estimation		Required sample size No. SAM cases (No. villages)			
Cluster sampling coverage survey	–	96 (46)	–	25.7% (17.6–33.7%)	–
Standard uncertainty in prior estimation (prior ±25%)					
Scenario 1a: broad program implementation^a^	55%	63 (30)	25.5% (15.4–34.6%)	34.7% (26.3–43.9%)	Strong (*p* = 0.0033)
Scenario 2a: basic program implementation^b^	52%	63 (31)	25.5% (16.0–34.4%)	33.8% (25.8–42.8%)	Strong (*p* = 0.0076)
Scenario 3a: external Implementation^c^	42%	62 (30)	25.4% (15.2–34.5%)	30.3% (22.5–39.6%)	Weak (*p* = 0.1165)
High uncertainty in prior estimation (prior ±35%)					
Scenario 1b: broad program Implementation^a^	55%	80 (39)	25.6% (18.2–31.6%)	30.0% (22.3–39.3%)	Strong (*p* = 0.021)
Scenario 2b: basic program Implementation^b^	52%	81 (39)	25.6% (18.2–31.6%)	29.6% (21.9–38.8%)	Strong (*p* = 0.0369)
Scenario 3b: external Implementation^c^	42%	79 (38)	25.7% (17.9–32.1%)	28.2% (20.8–37.3%)	Weak (*p* = 0.2582)

^a^Scenario 1: broad program implementation is the mean of six prior estimates (all with the exception of weighting and histogram provided by the external support team)

^b^Scenario 2: basic program implementation is the mean of four prior estimates including simple scoring, product of program performance, histogram of belief and previous SQUEAC coverage estimate

^c^Scenario 3: external implementation is the mean of two prior estimates including weighted scoring and histogram of belief by external support team

^d^The Z test is employed to test the null hypothesis of no conflict between the prior and likelihood coverage estimates

**Fig. 2:**
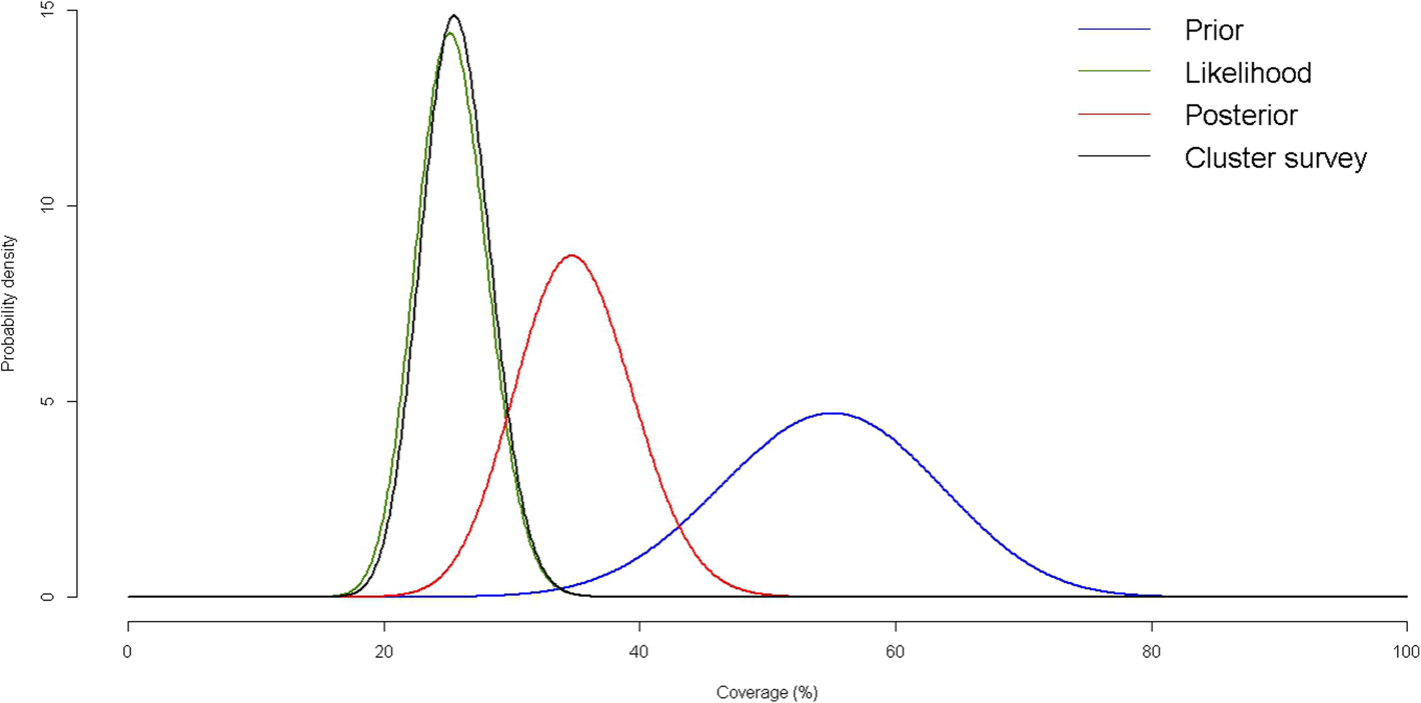
Coverage estimates from the two-stage cluster survey and SQUEAC conjugate Bayesian analysis with conflict (Scenario 1a).

Table 3 presents results of the two-stage cluster survey, as well as the SQUEAC under the alternative scenarios for prior estimation. The two-stage cluster survey yielded a final coverage estimate of 25.7% (95% CI: 17.6, 33.7%), with a total of 113 cases found, 29 of which were found to be undergoing treatment (either SAM or recovering from SAM). In the SQUEAC, prior estimates from the program staff (*Scenarios 1 and 2*) resulted in a conflict with the likelihood result, invalidating any interpretation of the resulting posterior estimate of coverage (for example Fig. 2). Even allowing for increased uncertainty, the conflict between the prior and likelihood data remained. The prior estimate produced by the external support team (*Scenario 3*) was 42% and was the only prior estimate that did not conflict with the likelihood data. The final coverage estimate from the external support team was 30.3% (95% CI: 22.5, 39.6%).

## Discussion

The SQUEAC methodology was developed to allow frequent evaluation of nutrition programs by program staff; the method produces qualitative insights into barriers to care and quantitatively estimates program coverage. In this analysis, we compare coverage estimates obtained through a two-stage cluster survey and the SQUEAC methodology. We found SQUEAC to be a technically demanding method for estimation which would appear to require a high level of objectivity possible with support from an experienced external or program team.

A limitation common to Bayesian methods, final coverage estimates can be biased if the prior estimate is inaccurate (i.e., very different from the true coverage proportion) and strong (i.e., with a narrow range of probable values), and if the sample size used for the likelihood survey is small (limited either by prevalence or the time and resources available). In these cases, the prior may dominate the analysis and result in a conflict that invalidates the final coverage estimate. Methods to develop a final prior estimate are presented in the technical guidance [8]. These methods are relatively simple to implement in program settings, but there is little evidence regarding the validity of their application in capacity-limited settings. Subjective or overly certain prior estimates produced from these methods may render the final coverage estimate invalid. More research to review and validate methods to produce accurate prior estimates may be needed.

Unfortunately, there is no way to a priori assess the accuracy of the prior, and this becomes evident only during formal testing for conflicts at the time of final analysis. In the case of conflict, current guidance instructs that the prior should be redefined in Stage 1 and Stage 2, and the Stage 3 likelihood survey conducted again. However, resource constraints rarely allow for this repetition. A practical alternative may be for the Stage 3 likelihood survey data alone to inform a final coverage estimate. The sample size for a Stage 3 likelihood survey, however, is small, and any resulting estimate would be less precise than desired. Therefore, when there is uneasiness around the prior development process, a larger likelihood sample size may be considered. The larger likelihood sample size would provide more precision to final estimates in the event that the final Bayesian estimate was not valid. While it is also recommended to increase the uncertainty around the final prior estimate (for example, from ±25% to ±35%) in such cases, doing so in this analysis did not prevent the resulting conflict.

To avoid the potential for biased estimation, implementers must be realistic about the position of the prior and the level of certainty. In our analysis, individual prior estimates were consistently higher than the population survey estimates of coverage, and estimates varied substantially by source. This is consistent with the potential for those intimately involved with the program (e.g., program staff and caregivers) to positively assess their own program, with recent evidence noting weighting and program assessment can be sensitive to the participant group [12]. Multiple sources of information should therefore be triangulated to improve judgment of the prior position, as described in the technical guidelines [8]. The use of multiple sources of information allows for broader perspective and community engagement; the combination of sources (weighted or unweighted) also may serve to counterbalance the limitations, subjectivity, or biases inherent in any one prior estimate to produce a more valid summary.

Our findings suggest several strategies that could ensure appropriate and quality implementation of the SQUEAC methodology. To begin, the selection of the appropriate method to describe program performance should depend on program needs and priorities. If qualitative information on barriers to access and a coverage estimate are required (both important for program assessment and improvement), the complete SQUEAC methodology may be implemented. The process of prior development may begin with review of relevant coverage estimates from similar programs or contexts. This may provide some external perspective and help orient initial estimates as the team gathers and triangulates additional secondary program data and qualitative information. If there is uncertainty in the prior, a larger likelihood survey may be allowed for to increase the precision of the likelihood estimate. This will increase costs but provide more protection against the possibility of not having a final coverage estimate to report if a conflict arises that invalidates the posterior coverage estimate in the conjugate Bayesian analysis. Implementation of the complete SQUEAC methodology with a larger likelihood survey may be particularly of interest in the first assessment of a new program, as the SQUEAC methodology ensures programs engage with communities and allows for a comprehensive understanding of community structures, communication, and health-seeking behavior that is critical for building an effective community mobilization strategy. If there is good certainty of the prior estimate, for example one which is informed by a quality SQUEAC assessment recently conducted, then the complete SQUEAC methodology with standard sample size for the likelihood survey may be appropriate. If only a quantitative estimate of coverage is required, or if there is doubt regarding the prior estimate produced during the SQUEAC Stages 1 and 2, it may be preferable to minimize influence of the qualitative and routine quantitative data review and shift limited resources to a two-stage cluster survey that uses a larger sample size to produce a robust coverage estimate. Lastly, if a coverage estimate is not a priority but qualitative information on barriers and boosters is more important for program monitoring and improvement, then a standalone qualitative assessment (e.g., SQUEAC Stages 1 and 2 or a community assessment) can be conducted.

While the choice in assessment design should be guided by program needs and priorities, it should also be noted that the two-stage cluster survey and SQUEAC require different resources, in terms of time, costs, and technical capacity. As coverage estimation in the SQUEAC requires development of a final prior estimate, a process that should be objective and iterative, correct implementation could require additional time and the engagement of higher-level program staff or external support. In settings where routine monitoring is well integrated into routine activities, and/or barriers and boosters of access are already well understood, the time needed to develop a final prior may be shorter. Qualitative data collected during Stage 1, however, may be useful for program improvement and warrant the additional commitment of resources and capacity in some settings.

Our study has some important limitations. First, we used a simple mean of individual prior estimates to calculate a single, final prior estimate. While this approach ensures that no individual prior estimate is used alone (as suggested in the technical guidelines), final prior estimation would ideally be a more iterative process that allows for reflection and revision of the final prior estimate with the critical evaluation of new information. The choice to calculate an unweighted mean of multiple individual priors resulted in a more linear approach but reflects common field practice. A weighted approach could also be adopted to assign relative importance to individual estimates for which there is more confidence; however, specific guidance on such methods is not yet available. Second, we present individual prior estimates developed by a range of participant sources, including local program staff and caregivers who may lack the necessary expertise, training, or objectivity to develop an accurate prior. The scenarios presented here, therefore, are not ideal but likely representative of pragmatic field conditions.

## Conclusion

Valid estimates of program coverage are needed to assess programs, inform allocation of resources, and improve evidence-based policy. Several methodologies are available to monitor program coverage, but methods differ in structure, priority, and cost. The overall SQUEAC methodology represents a step forward in the coverage assessment of nutrition programs by providing a means to both identify barriers to accessing care and estimate program coverage. We show that implementation of SQUEAC, however, can be technically demanding and requires the appropriate capacity during prior estimation to be informative. Program priorities and reporting requirements should inform the choice of study design, balancing feasibility and validity that are likely given available capacity.

## Additional file

Additional file 1: Supplementary Appendix. (DOCX 39 kb)

## Abbreviations

MSF: Médecins Sans Frontières
SAM: Severe acute malnutrition
SQUEAC: Semi-Quantitative Evaluation of Access and Coverage
