# Supplementary material for: Estimating program coverage in the treatment of severe acute malnutrition: a comparative analysis of the validity and operational feasibility of two methods
Source: Popul Health Metr. 2018 Jul 3;16:11. doi: 10.1186/s12963-018-0167-3 (PMC6029157; doi:10.1186/s12963-018-0167-3)
Supplement: Supplementary file 1 — Supplementary Appendix. (DOCX 39 kb) [file 12963_2018_167_MOESM1_ESM.docx]

Estimating program coverage in the treatment of severe acute malnutrition: a comparative analysis of the validity and operational feasibility of two methods

**Additional file 1**

SQUEAC Methodology

Stage 1 identifies patterns of program admissions, including areas of potentially high/low coverage, through analysis of routine program monitoring activities, beneficiary records, and readily available metadata. Qualitative information on negative and positive factors affecting coverage, perceptions of the program, and reasons for coverage failure is also gathered through informal group discussions and interviews with community and religious figures, caregivers, and program staff. Stage 2 evaluates hypotheses developed in Stage 1 related to spatial heterogeneity and reasons for coverage failure using case studies or small-area surveys in areas of suspected high and low coverage. Stage 3, conducted when an overall coverage estimate is desired and coverage is believed to be generally uniform (as indicated with a Stage 2 small-area survey and supporting qualitative and quantitative information from Stage 1), estimates coverage using a likelihood survey.

*Prior estimation*. The prior estimate used in the conjugate Bayesian analysis to inform estimation of overall coverage is intended to represent the range of current beliefs about the distribution of coverage using existing data and a range of opinions including external experts, program staff and /or community members (12).

Five methods are proposed in the technical guidelines (8) to translate analysis of qualitative and quantitative information gathered in Stage 1 and 2 into a prior distribution.

1) weighted scoring : All available information is synthesized into a set of positive (boosters) and negative (barriers) factors affecting access and coverage. Each positive or negative factor is listed and a weight (e.g. 1 for low importance to 3 points for high importance) that reflects its relative importance is applied to each. Weighting can be conducted among several participant groups (e.g. program staff or caregivers), who are asked to discuss evidence from Stages 1 and 2 and assign a weight to each factor taking into account prevalence, distribution of the factor, strength of the evidence and its potential impact on coverage. The sum of the weights of the positive factors and the negative factors are used to calculate a prior estimate using Equation 1.

$Prior mode=\frac{sum of positive factors+\left( 100-sum of negative factors \right)}{2}$ (Equation 1)

2) simple scoring: Each positive or negative factor suggested to affect coverage is listed as above and a fixed score is given to each. The sum of the scores of the positive factors and the negative factors are used to calculate a prior mode (Equation 1). In this method, the absolute number of positive and negative factors, but not the relative importance of each, is accounted for in the development of the prior estimate.

3) histogram of belief : all possible values of coverage value (0 to 100%, x axis) are discussed and the level of belief of whether each value is likely to be true (y axis) is collectively determined to create a histogram of belief of coverage.

4) product of program performance: quantitative estimates produced during Stage 1 are multiplied and deducted from the theoretical maximum coverage of 100% (Equation 2).

$Prior mode=100\%-(\% villages in service area$(Equation 2)

$$*\% cases identified through community case finding*\% cases refused admission *\% cases presenting promptly *\% cases not defaulting)$$

5) previous SQUEAC estimate: a previously reported SQUEAC estimate of coverage from the same program area may be directly used as a prior estimate.

All 5 methods above should ideally be used to develop a final prior estimate, but this will depend on time, resources and capacity of the assessment team. A minimum of 3 methods should be used to allow for triangulation. The final prior estimate is ideally generated using all available information using an iterative process that allows for reflection and revision and critical evaluation of new information. However, as is common field practice and in this analysis, the final prior estimate was generated by taking the simple average of the individual estimates produced in the various methods described above. No single method or source was individually used as a final prior estimate in the conjugate Bayesian analysis but rather used in combination with all other available information. Uncertainty around each final prior estimate, e.g. the minimum and maximum values for coverage that are consistent with prior information, is recommended as ± 25% of the mode (8).

In the conjugate Bayesian analysis, the mode, minimum and maximum values of the final prior estimate are used to calculate shape parameters (α and β) to describe the final prior probability density (Equations 3-4,(8)). This final prior probability density is combined with results of a Stage 3 likelihood survey to produce the posterior probability density of coverage. If the prior (Stage 1 and 2) and the likelihood (Stage 3) probability densities conflict (e.g. do not overlap), the posterior probability density should be treated with caution. The presence of a conflict between the prior and likelihood probability density is assessed using a Z test that provides evidence for or against the null hypothesis of no conflict between the prior and posterior coverage estimates.

$\alpha_{prior}= \mu* \frac{\mu*\left( 1-\mu\right)}{\sigma^{2}}-1$ (Equation 3)

$\beta_{prior}=\left( 1-\mu\right)* \frac{\mu*\left( 1-\mu\right)}{\sigma^{2}}-1$ (Equation 4)

where $\mu=\frac{prior minimum +4 *mode+prior maximum}{6}$ and $\sigma= \frac{prior maximum-prior minimum}{6}$.

*Sampling and data collection for SQUEAC likelihood survey (Stage 3) and two-stage cluster survey*

The local language used for describing malnutrition was used by the survey team in coordination with key community members to find all, or nearly all, current and recovering SAM cases in a sampled village. “Current cases” were defined according to the program admission criteria, as children aged 6 to 59 months with mid upper arm circumference < 115 mm, weight for height Z score <-3 (according to the World Health Organization Growth Standards) and/or bilateral pitting edema. When a case was identified, participation in the program was verified by asking the caregiver to show household stock of therapeutic food or program ration card. “Recovering cases” were defined as children currently enrolled in the program but no longer meeting the anthropometric criteria of a current case *and* not yet meeting the discharge criteria for the program.

The required sample size was translated into a minimum number of villages that must be sampled in order to find the required number of cases (Equation 2). Estimating the number of villages to be selected relies on often imprecise metadata and assumptions on average village size and population distributions. It therefore offers only a conservative estimate to help ensure the required number of SAM children can be identified, and the number of villages to be surveyed can be modified based on time and resource constraints.

$$n_{villages}= \frac{n}{average village size \times{\% population}_{6-59 months}\times SAM prevalence}$$

(Equation 5)

Using Equation 5 and assuming SAM prevalence of 1.7%, average village size of 681, and 6-59 month population of 18% (13), the 96 SAM cases required in the two-stage cluster survey translated into the selection of 46 villages.

*Statistical Analysis*

Single coverage (Equation 6) was estimated using the observed number of uncovered SAM cases, observed number of covered recovering cases and an estimate of uncovered recovering cases (11).

$single coverage= \frac{C_{in}+R_{in}}{C_{in}+R_{in}+C_{out}+R_{out}}$ (Equation 6)

where C_in_ = current SAM cases in the program, C_out_ = current SAM cases not in the program, R_in_= recovering SAM cases in the program, and Rout = recovering SAM cases not in the program estimated as $\frac{1}{k}\times(R_{in} \times\frac{C_{in}+C_{out}}{C_{in}}-R_{in})$ and k = the mean length of a recovered episode of SAM assumed to be 3 months.
